# CRAVAT: cancer-related analysis of variants toolkit

**DOI:** 10.1093/bioinformatics/btt017

**Published:** 2013-01-16

**Authors:** Christopher Douville, Hannah Carter, Rick Kim, Noushin Niknafs, Mark Diekhans, Peter D. Stenson, David N. Cooper, Michael Ryan, Rachel Karchin

**Affiliations:** ^1^Department of Biomedical Engineering, Institute for Computational Medicine, Johns Hopkins University, Baltimore, MD 21218, USA, ^2^In Silico Solutions, Fairfax, VA, USA, ^3^Center for Biomolecular Science and Engineering, University of California, Santa Cruz, CA 95076, USA and ^4^Institute of Medical Genetics, School of Medicine, Cardiff University, Heath Park, Cardiff CF14 4XN, UK

## Abstract

**Summary:** Advances in sequencing technology have greatly reduced the costs incurred in collecting raw sequencing data. Academic laboratories and researchers therefore now have access to very large datasets of genomic alterations but limited time and computational resources to analyse their potential biological importance. Here, we provide a web-based application, Cancer-Related Analysis of Variants Toolkit, designed with an easy-to-use interface to facilitate the high-throughput assessment and prioritization of genes and missense alterations important for cancer tumorigenesis. Cancer-Related Analysis of Variants Toolkit provides predictive scores for germline variants, somatic mutations and relative gene importance, as well as annotations from published literature and databases. Results are emailed to users as MS Excel spreadsheets and/or tab-separated text files.

**Availability:** http://www.cravat.us/

**Contact:** karchin@jhu.edu

**Supplementary information:** Supplementary data are available at *Bioinformatics* online.

## 1 INTRODUCTION

**Fig. 1.**
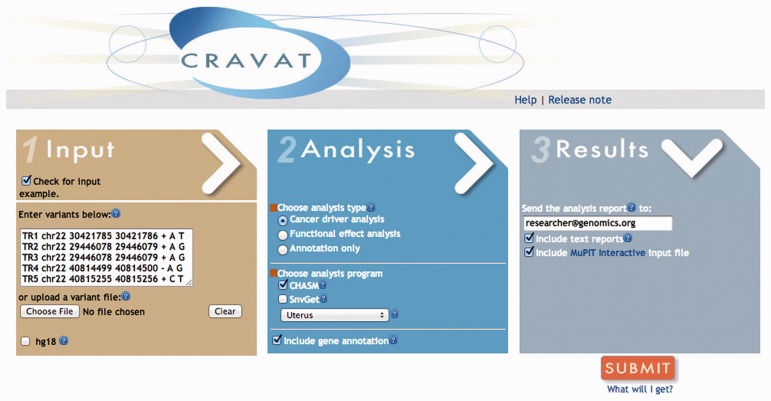
CRAVAT interface and workflow. (1) Input co-ordinates. (2) Select ‘Cancer driver analysis’, ‘Functional effect analysis’ and/or ‘Gene annotation’. (3) Results are delivered to the provided email address

With the advent of high-throughput sequencing technology, researchers face a bottleneck in terms of the time required to analyse the potential impact on disease aetiology of the many genetic variants routinely detected. Computational algorithms can in principle help researchers to prioritize and direct future experiments by narrowing down the numerous genetic alterations identified in sequencing studies. However, in practice, it can be challenging to run these algorithms in a researcher’s own laboratory, owing to the requirements of third-party software and databases, and large hard disk space and RAM specifications. We have developed Cancer-Related Analysis of VAriants Toolkit (CRAVAT), a web-based application that provides a simple interface to prioritize genes and variants important for tumorigenesis, allowing users to assess millions of variants in a single upload step (Fig. 1).

Numerous web implementations already exist for variant classifiers [reviewed in Karchin (2009)]. CRAVAT handles both germline and somatic variation but is dedicated to cancer genome analysis. It accepts variant calls from sequencing studies in either genomic coordinates (hg18 or hg19) or transcript coordinates—NCBI Refseq, CCDS and Ensembl (Pruitt *et al.*, 2007, 2009; Flicek *et al.*, 2012). Variants are mapped onto the best available transcript, using a greedy algorithm (see Supplementary Methods), and those variants that cause missense changes are identified. These variants can be scored in terms of their predicted impact on tumorigenesis, using the Cancer-Specific High-throughput Annotation of Somatic Mutations (CHASM) method (Carter *et al.*, 2009). They can also be scored by their predicted impact on protein function, with the Variant Effect Scoring Tool (VEST) (Carter *et al.*, 2013). Genes are ranked by their most significantly scored variant or mutation. Results are linked with published information from the 1000 Genomes Project (Clarke *et al.*, 2012), the Exome Sequencing Project, Catalogue of Somatic Mutations in Cancer (COSMIC) (Forbes *et al.*, 2008), GeneCards (Harel *et al.*, 2009) and PubMed, enabling users to compare predictions with known gene function, cancer associations and clinical/experimental studies. CRAVAT returns results *via* email in Excel and/or tab-separated text. It can also provide a formatted submission file for mutation Position Imaging Toolbox (muPIT) interactive (N.Niknafs *et al.*, submitted for publication), allowing users to visualize variants interactively in 3D, together with position-specific annotations.

## 2 SYSTEMS AND METHODS

CRAVAT runs on a Linux server with Apache Tomcat 6.0.35, and its web interface is written as Java Server Pages. When a user submits a job, a Java servlet is called, which places the job in the server’s queuing system, built on Redis backend and written in Python. When the queued job runs, a ‘master analyzer’ script written is launched to perform requested analyses, calling and processing the result of our prediction software and annotation utilities as needed. Local mirrors of annotation source databases are updated monthly. Prediction tools Single Nucleotide Variant Toolbox (SNVBox) (Wong *et al.*, 2011), CHASM and VEST are updated several times a year.

Depending on server load, run time for analysis of 1000 SNVs is ∼5–10 minutes. Run time scales linearly with the number of SNVs. A job with 1.8 million SNVs takes from 4 to 13 days. Benchmarking details are provided in the Supplementary Information. There is no limit to the size of a job. To ensure that large jobs do not hold up smaller jobs, jobs are separated into two queues, depending on size.

### 2.1 Prediction software

**CHASM:** Software to rank potential somatic driver mutations for specific cancer tissue types. It trains a classifier using parf, a fortran implementation of Random Forest (Amit and Geman, 1997; Breiman, 2001). The training set is a positive class of known cancer drivers from the COSMIC database and a negative class of simulated passenger mutations.

**VEST:** VEST scores variants by predicted protein functional impact. It also uses parf to train a Random Forest classifier. The VEST training set is a positive class of disease-causing germline variants from the Human Gene Mutation Database (HGMD Professional 2012v2) (Stenson *et al.*, 2009) and a negative class of common variants from the Exome Sequencing Project dataset (ESP6500 accessed July 2012) (http://evs.gs.washington.edu/EVS/]).

Both CHASM and VEST provide *P*-values and false discovery rate estimates to help the user establish a score cut-off for accepting predictions.

**SnvGet:** Returns 86 pre-computed features for each variant from the SNVBox database including the following: physiochemical properties of amino acid residues; scores derived from multiple sequence alignments of protein or DNA; region-based amino acid sequence composition; predicted properties of local protein structure; and annotations from the UniProtKB feature tables (UniProt Consortium and others, 2012). These features are used by CHASM and VEST to train classifiers and can be incorporated in new, user-generated predictive algorithms.

### 2.2 Annotation utilities

Each variant is annotated with database of single nucleotide polymorphisms identifiers, allele frequencies from the 1000 Genomes Project and ESP6500 populations, gene function information from the GeneCards database, the number of times that variant was observed in the COSMIC database and previous cancer association of the gene harbouring the variant, returned by PubMed search.

## 3 DISCUSSION

We provide an example to demonstrate how the CRAVAT web server can prioritize and facilitate mutation analysis. We obtained genomic coordinates of 184 824 mutations from The Cancer Genome Atlas sequencing study of 248 endometrial tumors from Firehose. We limited our submission to mutations that were called as ‘missense’ by Firehose, yielding 121 440 mutations. Options for ‘Cancer Driver Analysis’, ‘CHASM’, ‘Uterus’ tissue type and ‘Include gene annotation’ were selected. Results were received *via* email after 16 h: Excel spreadsheet with pages for ‘Variant Analysis’, ‘Amino Acid Level Analysis’ and ‘Gene Level Analysis’ and a separate text file to visualize amino acid substitutions in muPIT. On the ‘Variant Analysis’ sheet, 1066 mutations, of which 800 were unique, received a CHASM false discovery rate ≤0.3. Many significantly scored mutations were involved in pathways previously determined to impact endometrial cancer, e.g. PI3K, *Wnt* signalling, *MAPK* signalling and p53 signalling pathways (Kanehisa *et al.*, 2012). Several genes from these pathways (*PIK3CA*, *PTEN*, *TP53*, *KRAS* and *CTNNB1*) were known endometrial cancer driver genes (Liang *et al.*, 2012). In addition to identifying well-known drivers, CHASM identified potential drivers not previously associated with endometrial cancer, in biologically relevant pathways: viz *MTOR* in the PI3K pathway and *GSK-3B* in the *Wnt* signalling pathway).

### 3.1 Future work

CRAVAT is currently limited to analysis of missense mutations. We shall provide additional tools to analyse other types of mutation and to rank genes based on somatic mutation frequencies, aggregated *P*-values of CHASM or VEST scores, ratios of truncating to non-truncating mutations and counts of recurrently mutated positions. We also plan to include statistics useful in identifying which variant calls may be artifacts.

## Supplementary Material

Supplementary Data
