# Supplementary material for: CRAVAT: cancer-related analysis of variants toolkit
Source: Bioinformatics. 2013 Jan 16;29(5):647–8. doi: 10.1093/bioinformatics/btt017 (PMC3582272; doi:10.1093/bioinformatics/btt017)
Supplement: Supplementary Data [file supp_29_5_647__index.html]

CRAVAT: Cancer-Related Analysis of VAriants Toolkit — CRAVAT: cancer-related analysis of variants toolkit — CRAVAT: cancer-related analysis of variants toolkit — Supplementary Data 

# CRAVAT: cancer-related analysis of variants toolkit

## Supplementary Data

files

**Files in this Data Supplement:**

- Supplementary Data - xlsx file
- Supplementary Data - doc file
