# Supplementary material for: CRAVAT: cancer-related analysis of variants toolkit
Source: Bioinformatics. 2013 Jan 16;29(5):647–8. doi: 10.1093/bioinformatics/btt017 (PMC3582272; doi:10.1093/bioinformatics/btt017)
Supplement: Supplementary Data [file supp_btt017_Supplementary_Methods.doc]

# SuPPLEMENTARY METHODS

**Transcript Selection:** For the purpose of mapping missense mutations onto transcripts, we developed an algorithm to select a "best transcript". First, protein-coding transcripts from RefSeq (Pruitt *et al*., 2007) and Ensembl (Flicek *et al*., 2012) are clustered into loci (sets of overlapping transcripts on the same DNA strand) using the *clusterGenes* tool from the Kent Source library (Drezner *et al.,* 2012) (Suppl. Fig. 1).

**Suppl. Figure 1. Refseq and Ensembl transcripts for a single cluster returned by the clusterGenes software.** This locus corresponds to the gene *IL18BP* and has seven RefSeq transcripts and thirteen Ensembl transcripts. The algorithm drops those transcripts that do not contain any coding bases (*e.g.* ENST0000414358, ENST0000525932, ENST0000531777). Image created with UCSC Genome Browser (Kent *et al.*, 2003).

Next, each locus is divided into blocks. Blocks are defined only for the CDS (coding sequence). Bases that reside within the 5' UTR or 3' UTR are excluded. Within the CDS, a new block is initiated when an exon start or exon stop occurs in any transcript. Blocks capture instances of overlap across the locus (Suppl. Fig. 2).

**Suppl. Figure 2. The CDS region of the locus is divided into twelve blocks.** Blue=transcripts from RefSeq. Red=transcripts from Ensembl. Blocks are defined by the occurrence of an exon start or stop in any of the transcripts. Image created with UCSC Genome Browser (Kent *et al.*, 2003)

Each transcript was then scored with a metric that prioritizes the coverage of coding bases and agreement between RefSeq and Ensembl transcript definitions.

Let *t_i_* represent the *i^th^* transcript, where *i$\in${1, ... , n}* and b*_j_* represent the *j^th^* block, where *j$\in${1, ... , m}.*  *l_j_* is the number of coding bases in *b_j_*. Since all transcripts are derived from the same DNA strand, they can occur in one of three possible reading frames. Let *r(t_i_) $\in${0,1,2}* be the reading frame of *t_i_ .* Then the score of the *k^th^* transcript *t_k_* is

$S(t_{k})=\frac{1}{n}\sum_{j=1}^{m} l_{j}\sum_{\begin{matrix} i=1, \\ i\neq k \end{matrix}}^{n} \delta\left(r(t_{k}),r(t_{i})\right)$ Supp Equation 1

where $\delta(x,y)$is the Kronecker delta function:

$$\delta(x,y)=\left\{\begin{matrix} 1\text{ if }x=y \\ 0\text{ otherwise} \end{matrix}\right.$$

We use a greedy algorithm to select a single "best" transcript for any position. First, transcripts with a frameshifted exon are removed. Then all remaining transcripts are scored (Suppl. Equation 1) and the lowest scoring transcript is dropped and the scores recalculated. These steps are repeated until no "frameshifted" transcripts (those that include an exon frameshifted relative to the consensus reading frame) remain. The consensus reading frame is determined by majority vote. When the procedure terminates, the final scores of all transcripts that remain are returned and the transcripts are ranked.

**Suppl. Figure 3. Transcripts scored with a greedy algorithm.**  Final scores of transcripts from Suppl. Figs. 1 and 2. Green = Transcripts that were "Keepers" because they match the consensus reading frame. Red=Transcripts that were discarded because they were defined as frameshifted (coding exon 5 is in a different reading frame from the consensus reading frame). Image created with UCSC Genome Browser (Kent *et al.*, 2003)

In Suppl. Figure 3, there is a nine-way tie for best scoring transcript (best score is 0.731). However, the nine transcripts differ from each other only in UTR regions. Thus, any one of these transcripts would be an equally good choice of "best available transcript" and we randomly select one. In this case, "NM_001145057" was chosen.

Mutations are mapped onto the highest ranking transcript that includes the exon with the mutation.

This process was designed to give maximum coverage of the exome, while prioritizing those transcripts whose reading frames concur with the majority of available transcripts from both RefSeq and Ensembl.

Although many users of CRAVAT may prefer to have a single transcript selected, other users may prefer to see all available transcripts. We shall make this option available in a future release of CRAVAT.

References

Dreszner, T.R. *et al.* (2012). The UCSC genome browser database: extensions and updates 2011. *Nucleic Acids. Res.,* **40*,*** D918-923.

Flicek, P. *et al.* (2012). Ensembl 2012. *Nucleic Acids. Res.,* **40*,*** D84-90.

Kent, W.J. *et al.* (2002). The human genome browser at UCSC. *Genome Res.,* **12**(6):996-1006.

Pruitt, K.D. *et al*. (2007). NCBI reference sequences (RefSeq): a curated non-redundant sequence database of genomes, transcripts and proteins. *Nucleic Acids Res*.*,* **31**, 3812-3814.
